# The Role of an Individual and a Situation in Explaining Work Addiction: Disclosing Complex Relations

**DOI:** 10.3390/ijerph20054560

**Published:** 2023-03-04

**Authors:** Modesta Morkevičiūtė, Auksė Endriulaitienė

**Affiliations:** Department of Psychology, Faculty of Social Sciences, Vytautas Magnus University, 44191 Kaunas, Lithuania

**Keywords:** work addiction, personality, work motivation, parents, organization, latent profile analysis, moderated mediation

## Abstract

The current study aimed to test the relationships between perfectionism, type A personality, and work addiction via mediator of extrinsic work motivation and moderators of both parent work addiction and demanding organization profiles. A cross-sectional study was carried out using an online self-report questionnaire. A sample consisted of 621 employees working in different Lithuanian organizations that were selected on the basis of the convenience principle. Prior to testing the hypotheses, latent profile analysis (LPA) was conducted in order to identify the subgroups of participants based on situational variables. Two profiles (i.e., ‘less addicted parents’ and ‘more addicted parents’) for parent work addiction and tree profiles (i.e., ‘slightly demanding organization’, ‘moderately demanding organization’, ‘highly demanding organization’) for a demanding organization emerged from LPA. The hypotheses were tested using structural equation modeling. Main results revealed that direct relationships between perfectionism, type A personality, and work addiction were positive and stronger for those working in highly demanding organizations. Indirect relationships between perfectionism, type A personality, and work addiction (via extrinsic motivation) were positive and stronger for employees who have parents with higher levels of work addiction. Future researchers and those who implement preventive practices should be aware that personal factors can be the first impetus for work addiction, and the second one (comprised of situational factors in a family and organization) can enhance the expression of these personal factors and stimulate the development of work addiction.

## 1. Introduction

Work addiction has become increasingly prevalent addiction in the modern workforce, which is linked to long-term negative repercussions [1]. Those who are addicted become so immersed in their work due to their inner drive to work excessively that they struggle to detach themselves from it, ultimately neglecting their need for recovery [2]. It is not surprising, therefore, that work addiction may lead to severe implications for employee health and general well-being in a form of exhaustion [3], occupational stress, job burnout [4], psychological distress [5], depressive mood, disabling back pain [6], sleep problems, cardiovascular risk [7], etc. Other authors [8] note poor family relationships, marital dissatisfaction, and dysfunction in the family, which are also truly likely consequences considering the fact that those addicted to work spend an exorbitant amount of time working at the expense of their family obligations. Finally, even though work addiction is frequently considered an ‘acceptable addiction’ by some employers and is often regarded as model behavior [9], erosion of employee energetic capacity actually results in reduced quality of work, which indicates negative effects of work addiction on the organizational level too [10]. 

Previous research suggested that work addiction should be prevented as much as possible, as it leads to more disadvantages than advantages [11]. One of the remedies helping realize health promotion of workers is limiting the development of the issue. Against this background, researchers focused on analyzing contributors that may serve as a strong indication of work addiction prediction. However, there are still important limitations to address in this line of research. 

Studies on work addiction attribute great explanatory power to the trait theory [12]. Therefore, many papers (e.g., [13,14,15,16]) on the predictors of work addiction are focused on personal factors (i.e., mostly personality traits) as primary contributors. However, this perspective suffers from an oversimplified approach because it considers compulsive conduct toward work apart from contextual factors [12]. Theoretical models [17,18,19] assume that work addiction is in fact a complex phenomenon induced by both personal and situational/contextual factors and that none of the perspectives individually can fully explain work addiction. Fragmentary examination of work addiction does not allow exhaustively revealing development processes or the interactions between important contributors, restricts a comparison of the factors determining work addiction, and limits the disclosure of the most important predictors within the context of other possible contributors. That is, studies that do not analyze the effect of different sources of influences may be regarded as producing a relatively incomplete and limited explanation of the phenomenon in question. Unfortunately, it is this type of research that is most common in the field of work addiction.

To address the above issues, the current study, for the first time, tested an integrated model comprised of all the sources of influences that have been recognized to be major ones in predicting work addiction (i.e., personality, family, and organization) [20]. By doing this, we presented a broad and exhaustive picture of how work addiction may develop, which assists in improving preventive practices and provides knowledge of which exact aspects to focus mostly on in order to limit the escalation of work addiction. We used the previously unexplored theoretical model of Liang and Chu [17] as the basis for formulating our hypotheses. In the present study, we revised this model by incorporating the most recent results and extended the originally proposed [17] interaction line of moderation by adding the aspect of mediation through motivational forces hereby going beyond prior research and taking a step further towards a more nuanced framework of work addiction predictors. Alongside the traditional variable-centered approach, the person-centered strategy was used in the present study, which provided a more holistic viewpoint and made our study even more appealing. 

Specifically, the present research aimed to examine the relationships between perfectionism, type A personality, and work addiction via mediator of extrinsic work motivation and moderators of both parent work addiction and demanding organization profiles. To fulfill this aim, first of all, we present a review of relevant literature in the following sections. Then, we describe the sample of our study and measurements used. At the end of the paper, the results of the empirical investigation and concluding remarks are discussed. 

### 1.1. Work Addiction

Since the first time Oates [21] presented the focal phenomenon of our study, scholars have made attempts to conceptualize it from various perspectives. Unfortunately, no clarity has been reached on its definition so far. One of the main reasons for this is related to the construct contamination by the overlapping phenomena [22]. For instance, a scientific debate on the juxtaposition of work addiction with workaholism is currently ongoing. An interchangeable use of these terms without a clear and refined perspective led to the considerable confusion posing a threat to the reliability of research results. Against this background, very recently, a quantitative literature analysis was performed [23], which produced the first evidence for the assumption that work addiction and workaholism are conceptually and empirically distinct forms of excessive work behavior. Workaholism was found to be a more generic phenomenon that included a wider range of theoretical underpinnings and was specified by the general aspects of compulsive and excessive working, whereas work addiction was theoretically rooted in addiction literature and was characterized by clinical criteria that are common to all addictions [23]. Since work addiction was noted to be much more explicit [23], a decision was made to analyze particularly this construct in the present study.

Given that most conceptualizations on the phenomenon were not based on a well-defined theoretical foundation and did not comprise the core aspects of addictions [24,25], an improved conceptualization was proposed [26], which was used throughout this paper. Work addiction was defined as being overly concerned about work, driven by an uncontrollable work motivation, and spending so much energy and effort on work that it impairs private relationships, spare-time activities, and/or health [26]. Anchored in general addiction theory and based on the addiction components model [27], the conceptualization employed in the present paper operationalized work addiction as a set of seven elements: (1) salience (working activity dominates thoughts and behavior); (2) mood modification (work is used as a way to modify mood); (3) tolerance (increasing amounts of work are required to achieve the former effects); (4) withdrawal (occurrence of unpleasant feelings when unable to work); (5) conflict (working activity comes in conflict with other spheres of individual’s life); (6) relapse (reversion to earlier patterns of excessive working after abstinence or control); (7) problems (excessive working negatively affects health and causes other problems for a person) [26]. 

Research dealing with the origin of work addiction have allowed it to be perceived as a consequence of both personal and situational factors [20]. However, literature where these factors are examined together to disclose their relationships within the context of work addiction is scant. Since a more comprehensive view of the onset of work addiction is desirable and recommended [10], starting from the following section, we build argumentation for the empirical investigation of collective influences.

### 1.2. The Research Model

There are several central theories explaining what drives people into work addiction. The paradigms employed to date include addiction [28], learning [29], trait-based theories [30], and, more recently applied, cognitive frameworks [31], along with motivational theories [32], family-system [33], and organizational perspectives [34]. Although most of these perspectives have relevance to addictive behavior, this relevance often remains limited to certain issues or conditions. One general problem seems to be that the majority of these perspectives start out from a too narrow-band theory of human activity. They focus on specific agents of limited effects ignoring the fact that many different factors play a role in maintaining the addiction trap. 

In our view, it is necessary to have an integrated framework that comprises different perspectives. It was for this reason that we set out to search for a comprehensive model of work addiction. However, truly comprehensive models are actually hard to find. There have been only few multiple-variable integrative theoretical models on the acquisition of work addiction (i.e., [17,18,19]) and only one of them [17] offered well-described measurable relationships among those multiple factors, thereby demonstrating its advantage over other models. Given the fact that the model proposed by Liang and Chu [17] has not been tested yet, it was used as a basis for formulating our hypotheses.

The authors [17] emphasized that the factors of perfectionism, obsessive compulsion, achievement orientation, and conscientiousness were key elements responsible for fueling work addiction. Alongside the latter, organizational encouragement to put work before family, peer competition in a workplace, vicarious learning in the organization, as well as in the family, and intrinsic work values were also claimed to play an important role in predicting work addiction. Hence, the model suggested by Liang and Chu [17] covered all the sources of influences recognized to be critical ones in the context of work addiction (i.e., personality, family, and organization) [20].

Nevertheless, after careful consideration of the aforementioned model within the context of the current work addiction studies, we believe that it should be revised to include advanced knowledge. Therefore, in the present study we propose to update this model including the most recent empirical evidence in it. For this purpose, we relied on a recently performed literature review [35], which examined the factors that predisposed people to become work addicted. Specifically, variables of extrinsic work motivation, the workload, interpersonal conflicts, and the role conflict at work were incorporated. In addition, several modifications to original model were made. Since the latter encompassed certain highly debatable components (i.e., conscientiousness, intrinsic work values) [8,36], as well as some rarely explored and overlapping variables (i.e., obsessive compulsion) that could be covered under broader factors of a higher prognostic power in a model, a decision was made to further investigate the model without taking these variables into consideration.

**Figure 1:**
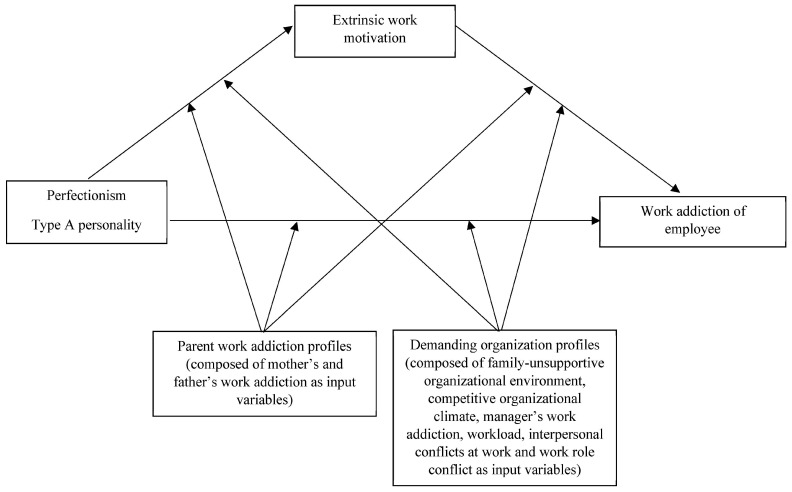
Theoretical moderated mediation model.

To sum up, a revised work addiction model (see Figure 1) was adjusted at the level of discrete components but retained an original structure of the main sources of influences. The model comprised a set of measurable elements since we supplemented it only with the measurable factors, whereas Liang and Chu [17] proposed directly observable alternatives for the replacement of abstract concepts (i.e., achievement orientation, encouragement to put work before family, peer competition, vicarious learning at the workplace and in the family) in a model. Hence, the source of personality reflected in such variables as perfectionism (a personality trait characterized by striving for flawlessness and setting high standards of performance accompanied by tendencies for overly critical evaluations of one’s behavior [37,38]) and type A personality (a set of impatience, ambitiousness, competitiveness, and hostility [39]). The second source that we named ‘parent work addiction’ included the perceived work addiction of both mother and father (for the definition see previous section). The third source that we named ‘demanding organization’ included the perceived factors of family-unsupportive organizational environment (the environment that encourages workers to devote themselves to their work at the expense of other life domains (i.e., family) [40]), the competitive organizational climate (the comprehension of organizational rewards to be dependent on comparing employee performance with that of his/her coworkers [41]), work addiction of a manager (for the definition see previous section), the workload (the sheer volume of work required of an employee [42]), interpersonal conflicts at work (disagreements between coworkers in the workplace [42]), and the work role conflict (the perceived demand to take on inconsistent or incompatible job tasks [43]). Since the variable of extrinsic motivation defined as a set of energizing forces for the purpose of achieving the desired outcome, which initiates work-related behavior and determines its form, direction, intensity, and duration [32,44], did not fall under any of the aforementioned groups of factors, we separated it from other variables in a model, thus forming an autonomous source of influences. 

### 1.3. Relationships Explaining Work Addiction

The more elements are included, the more possible interrelations can be examined. We have already presented several of them in other papers by testing indirect relations between parent work addiction [45], demanding organization [46], and employee work addiction via work motivation as the mediator. In the present paper, we took steps in a similar direction by examining the motivational mechanism of work addiction too. However, following the propositions of Liang and Chu [17], the initiator of this motivational mechanism was different herein. Since the personality was found to be a key threat to work addiction with the greatest prognostic power among all other risk factors [8], in the present paper it was placed in a leading position for triggering the motivational mechanism of work addiction. Further, with the guidance of Liang and Chu [17], the moderation aspect was investigated as well. Contrary to personality, family- and organization-related factors were traditionally considered to be secondary agents, performing more transitory and situational roles [20]. Hence, parent work addiction and demanding organization were assumed to play moderating roles within our research model. In this way, the moderation and mediation were combined into a single examination (see Figure 1), which enabled us to sensibly organize the multiple sources of influences, to maintain the original notions [17], and to try the new ones at the same time. To make our reasonings more solid, in the following paragraphs we present a detailed argumentation on both indirect relationships that address the question of ‘how work addiction arises?’ and possible conditioning effects that solve the issue of ‘for whom this indirect relationship holds true?’.

Before moving to more elaborated relations, Liang and Chu [17] presented the central links that laid the groundwork for their subsequent reasoning. Basing their arguments on the trait perspective, the authors [17] started with the assumption that a personality was an essential contributor predicting work addiction. Since perfectionism and type A personality were considered to be one of the strongest personal correlates within the work addiction context [8], Liang and Chu [17] assumed them to pose the highest risk for this particular addiction to arise. There is actually some ground for this assumption. On the one hand, perfectionism is more characterized by orientation to improvement [37], whereas type A personality reflects the go-getter attitudes [47], which indicates differences between the two. On the other hand, they have also notable commonalities since both perfectionism and type A personality guide the behaviors in very similar directions. More specifically, perfectionism and type A personality are both associated with reaching the perceived best result [8,48]. Perfectionists and those having type A personality seem to be greatly concerned and rigid about the performance outcomes [49,50]; staying at the middle level is far from what is in target of such individuals [8]. All this clearly directs an individual to work because the latter is an obvious tool that can serve well in achieving the best. Considerable efforts devoted to work maximize chances for a successful outcome and may protect one from being a poor performer (that perfectionists and those having type A personality seem to be in fear of [51,52]). As a result, individuals keep on working until the feeling of ‘enough’ is reached [53]. However, due to the persistency, which is a feature of perfectionists and type A personality individuals [8,54,55], it is possible to simply fall into an endless process of excessive working with severe difficulties to withdraw from work. Hence, both perfectionism and type A personality pose a risk to work addiction mainly because they are characterized by establishment of high-performance aspirations, which shows a straightforward direction towards work. Based on the abovementioned reasoning, we put forward the first hypothesis:

**Hypothesis** **H1:** *Employee perfectionism and type A personality are related to increased work addiction.*

Since personality is believed to impact work-related behaviors mainly through motivational processes [56] and because extrinsic motivation is characterized by the features (i.e., achieving a desired consequence) which form the basis for driving work addiction [28], in the present study we suggest it to operate as the mediator explaining why or how perfectionism and type A personality pose a work addiction risk. Extrinsic motivation could be considered a serious candidate for explaining these relations because it plays an important role in effect transmission. Specifically, conditioned by general orientation towards the best possible outcome, perfectionism and Type A personality transmit themselves into a strong desire to pursue performance goals [57,58]. Satisfaction with accomplishing these goals further strengthens the motivation and simultaneously encourages one to continue working as a necessary element for performance goals to be achieved [59]. This forms a trap for work addiction, which is a likely consequence stemming from highly motivated long-lasting repetitive working behavior [60]. With this in mind, we propose the following hypothesis:

**Hypothesis** **H2:** *Employee perfectionism and type A personality are related to increased work addiction through higher levels of extrinsic work motivation.*

Further, taking into account a relevant contribution of the authors [17] in detailing complex relationships, we retained the originally proposed moderating effects in a revised model. Liang and Chu [17] suggested that high levels of family and organizational inducements served as catalysts for direct relationships between perfectionism, type A personality, and work addiction. There is a plausible explanation that could underlie these effects. Given that the most obvious feature of work addiction is excessive work, those prone to work addiction, first and foremost, need to perceive or believe that they have to perform hard before they overinvest in working [61], and the fact of having work-addicted parents, as well as working in demanding environments, may be assumed to be at least partially responsible for that. For instance, demanding organizations generate elevated expectations in terms of employees’ productivity [62] and, therefore, those working under demanding organizations may feel compelled to work hard [63]. Similarly, those having work-addicted parents are pushed to live up to the perfectionist standards and, accordingly, to fulfill the external requirements in a form of highest achievements [64]. Hence, those having work-addicted parents and belonging to demanding organizations receive clear signals about certain behavioral norms and may perceive this as a pressure to spend much time and effort on work. This may activate the addictive traits that are the focus of the present study and limit people in terms of possible behaviors through which their underlying tendencies toward work addiction can be expressed, thus creating the most favorable conditions for this particular addiction to develop [17].

Our reasoning, however, does not end at this point. In a very general sense, the facts of working in a demanding organization, as well as having work-addicted parents, may exert a great impact on the outcomes at a motivational level too. Therefore, a possibility arises for the moderation of the above-discussed indirect relationships depicted in previously formulated H2. More specifically, by activating the expression of addictive traits, situational factors analyzed in the present study simultaneously restrict people not only in their behaviors but also in the reasons for these behaviors [45,46]. This is mainly because those having work-addicted parents and belonging to demanding organizations not only receive signals about the behavioral norms but are also offered important extrinsic compensatory benefits to keep to those norms [45,46,64,65]. Hence, it is reasonable to expect the indirect relationships between perfectionism, type A personality, and work addiction (through extrinsic motivation) to show some situational specificity. 

Before formulating the hypotheses that are in line with our reasoning, we would like to draw attention to the methods used in the present study. The majority of researchers on the onset of work addiction has relied on a variable-centered approach where the focus is on the associations between the variables. An alternative to the traditional variable approach is to adopt a person-centered approach where an individual is the unit of analysis. The person-centered approach is known to be better for testing a set of the observed measures as it allows studying them together, as a whole [66]. Therefore, alongside the variable-centered approach, we used person-centered strategies (specifically latent profile analysis (LPA)) and assumed that the associations within the multiple-variable groups of situational predictors can be explained by the existence of latent profiles. Following well-established practices in person-centered research and considering the explorative nature of LPA, we were cautious about formulating potential profiles precisely. Moreover, the studies (e.g., [67,68]) that looked at naturally occurring profiles of participants in the fields we are interested in are scarce and unsuitable for making precise assumptions since the criteria variables used were not completely the same as those in our study. As a result, only very general presumptions could be made. Based on visible tendencies of previous studies [67,68], we expected to find at least high- and low-level profiles (with high and low scores across all or most measures) for parent work addiction and a demanding organization separately. We assume these profiles to be critical ones in the hypothesized relations. Hence, our assumptions are as follows:

**Hypothesis** **H3a:** *Direct relationships between employee perfectionism, type A personality, and work addiction are moderated by* *parent work addiction profiles. These relationships are stronger for employees in a high-level profile (as compared to those in a low-level profile).*

**Hypothesis** **H3b:** *Direct relationships between employee perfectionism, type A personality, and work addiction are moderated by demanding* *organization profiles. These relationships are stronger for employees in a high-level profile (as compared to those in a low-level profile).*

**Hypothesis** **H4a:** *Indirect relationships between employee perfectionism, type A personality, and work addiction via extrinsic work motivation are moderated by* *parent work addiction profiles. These indirect relationships are stronger for employees in a high-level profile (as compared to those in a low-level profile).*

**Hypothesis** **H4b:** *Indirect relationships between employee perfectionism, type A personality, and work addiction via extrinsic work motivation are moderated by demanding* *organization profiles. These indirect relationships are stronger for employees in a high-level profile (as compared to those in a low-level profile).*

## 2. Materials and Methods

### 2.1. Participants

A total of 1164 individuals working in different Lithuanian organizations (e.g., public hospitals, educational institutions, private service, as well as sales organizations, etc.) agreed to participate in the study. However, a part of the initial sample was eliminated in order to ensure data quality. Such a decision was made because some the participants were able to provide assessment of several constructs (i.e., their parents’ working behavior) based only on their memory rather than on the current case. Since different conditions under which the participants evaluated their parents resulted in measurement contamination, only those individuals were included whose both parents were working at the time the study was conducted.

Thus, the final sample consisted of 621 individuals. The participants were predominantly females (71%). The age ranged from 18 to 62 years (*M* = 29.35 years; *SD* = 8.43). The majority of the participants (81%) had higher education. The average job tenure at the present workplace was 4.43 years (*SD* = 6.07). As to the job sector, 62% of the participants were employed in the private sector; 38% were employed in the public sector.

### 2.2. Data Collection

Several ways (e.g., individual invitations, a public invitation in social media) were used to ask individuals to participate in the study. The main part of the participant sample, however, was invited by the administration or top executives of organizations that were selected on the basis of the convenience principle. Specifically, organizations were sent an e-mail invitation containing the main information of the investigation. The organizations that were interested in participating were sent an online survey link with a request to forward the invitation message to the members of the organization. The completed questionnaires were delivered directly to the dataset of the survey platform ‘Google Forms’ that was accessible to the authors of the present study only. 

Data were collected with the help of an online self-report questionnaire. The participants provided responses based on their experiences or perceptions. The approximate duration of the survey was 20 min. 

### 2.3. Informed Consent

Before being given access to the survey, the participants had to confirm that they had become acquainted with and understood the informed consent. The most important thing was that information about the aim of the study, anonymity and confidentiality of the responses, and how the data would be used was provided. The form also explained that participation was voluntary; those who completed the questionnaire were not monetarily compensated. The participants were ensured that their option to withdraw from the study at any time would be respected and that they had the right to revise and change their answers. 

### 2.4. Measures

To test the hypotheses, several well-established previously validated instruments were used. All the instruments that had not been used for Lithuanian samples before (i.e., those measuring work addiction, type A personality, a work role conflict, a competitive organizational climate, the family-unsupportive organizational environment) were back-forward translated into Lithuanian. For information on psychometric properties of all the scales see Section 3.1. 

Work addiction. The Bergen Work Addiction Scale (BWAS) [26] was used to measure work addiction. BWAS is composed of seven items all reflecting seven core elements of addiction that have manifested themselves during the past year (e.g., ‘How often did you deprioritized hobbies, leisure activities and exercise because of your work last year?’). We used BWAS to measure work addiction of the employees, their parents, and immediate managers. The employees were asked to evaluate their own, supervisors’, as well as their parents’ (mother’s and father’s separately) work addiction. We used specific filter questions to control such aspects as having an immediate supervisor, as well as being well acquainted with parents’ work behaviors. Considering the fact that the employees could encounter some difficulties when evaluating work behaviors of their parents and managers during the past year, we asked the participants to give general answers with no time boundaries (e.g., ‘How often does your mother deprioritize hobbies, leisure activities, and exercise because of her work?’). The items were rated on the five-point Likert scale (from 1 (never) to 5 (always)). BWAS provides both continuous scores and categorization of work addiction [26]. Only continuous scores were used in the current study. Hence, a higher result indicated a more expressed work addiction experienced or perceived by the participant. 

Extrinsic work motivation. Extrinsic work motivation was assessed using the 12-item subscale that was taken from the general Work Extrinsic and Intrinsic Motivation Scale (WEIMS) [44]. All participants were presented with various reasons for performing work that reflected extrinsic motivation (e.g., ‘Because I want to succeed at this job, if not I would be very ashamed of myself’). We asked them to indicate to what extent each of these causes corresponded to the reasons for their present involvement in work on the seven-point Likert scale ranging from 1 (does not correspond at all) to 7 (corresponds exactly). A higher score represented a higher level of extrinsic work motivation of the participant. 

Perfectionism. To measure perfectionism, a short version of the Multidimensional Perfectionism Scale (MPS) [38] was used. The short version was employed because it showed alpha reliabilities comparable to the full version but better factorial validity [69]. The scale captures self-oriented perfectionism (characterized by setting exceedingly high standards for oneself), other-oriented perfectionism (characterized by holding perfectionistic standards for others), and socially prescribed perfectionism (characterized by beliefs that significant others have high standards for an individual) [38]. However, in the present study, only two dimensions of perfectionism were measured (i.e., self-oriented perfectionism and socially prescribed perfectionism) because other-oriented perfectionism was not considered to be the core dimension of overall perfectionism [70]. The scale contained a total of 10 items. The sample item was ‘One of my goals is to be perfect in everything I do’. The items were rated on the seven-point Likert scale ranging from 1 (strongly disagree) to 7 (strongly agree), with higher scores indicating greater levels of overall perfectionism expressed by the participant.

Type A personality. The 10-item Framingham type A personality scale (FTAS) [39] was used to assess type A personality. The participants were asked to indicate to what extent the listed aspects (e.g., ‘Being hard-driving and competitive’) are inherent to their case or describe them. The items were rated on the four-point Likert scale ranging from 1 (not at all) to 4 (very well), with higher scores indicating a more expressed type A personality.

Family-unsupportive organizational environment. To identify perceptions that the participant forms regarding the extent to which the organization is family-(un)supportive, the Family-Supportive Organization Perceptions Inventory (FSOP) [40] was used. The participants were preceded by instructions to evaluate the philosophy of the organization. The sample item was ‘It is assumed that the most productive employees are those who put their work before their family life’. The 14-item inventory was rated on the five-point Likert scale ranging from 1 (strongly disagree) to 5 (strongly agree). We reverse-coded several items so that higher scores of the scale indicate negative perceptions towards an organization’s support for balance between work and family.

Competitive organizational climate. A competitive organizational climate was measured with a four-item scale that was initially proposed by Brown and colleagues [41] and further adapted to a wider population by Fletcher and colleagues [71]. The sample item was ‘My coworkers frequently compare their performance with that of mine’. A seven-point response scale ranged from 1 (strongly disagree) to 7 (strongly agree). A higher score indicated a higher level of the perceived competitive organizational climate. 

Workload. The five-item Quantitative Workload Inventory (QWI) [42] was employed to measure workload. The scale assessed the participants’ perception of work in terms of volume and pace. The example item was the following: ‘How often is there a great deal to be done?’. Five response alternatives were given ranging from 1 (less than once per month or never) to 5 (several times per day). High scores represented a high level of perceived workload. 

Interpersonal conflicts at work. The Interpersonal Conflict at Work Scale (ICAWS) [42] was used to assess disagreements in the workplace. The ICAWS includes four items referring to the frequency of arguments in the workplace and a rude behavior of co-workers (e.g., ‘How often do other people yell at you at work?’). The instrument has a five-point response scale ranging from 1 (less than once per month or never) to 5 (several times per day). High scores represented frequent conflicts at the workplace. 

Work role conflict. The eight-item Role Conflict Scale [72] was used to measure a work role conflict. The sample item was ‘I have to do things that should be done differently’. The participants were asked to respond to the items on the seven-point Likert scale ranging from 1 (very false) to 7 (very true). A higher score indicated a higher level of a work role conflict perceived by an employee.

Finally, based on previous research, socio-demographic variables could show significant differences in focal variables of the present study [73] and, therefore, may be assumed to affect the hypothesized relations to some extent. Therefore, we also gathered a set of variables describing the socio-demographic characteristics of the sample. Specifically, the participants were asked to provide information about their gender, age, education, working tenure in the present workplace, as well as the sector they worked in.

### 2.5. Statistics

The statistical analysis was performed on SPSS 23.0, AMOS 23.0, and Mplus 8.8. It consisted of several stages. First, to verify the convergent validity and reliability of the constructs, the confirmatory factor analysis (CFA) with structural equation modeling (SEM) was implemented. Alongside χ^2^ goodness-of-fit statistics, several previously recommended [74] indices were used to indicate a structural equation model fit: Comparative Fit Index (CFI), Tucker-Lewis Index (TLI), Root Mean Square Error of Approximation (RMSEA). We considered the values close to or higher than 0.95 for CFI and TLI and the values close to or lower than 0.06 for RMSEA to be a good fit [74]. The parameters were estimated using maximum-likelihood (ML) estimation. In order to verify discriminant validity, a correlation matrix among the items was generated. In addition, a partial correlation analysis controlling the participants’ socio-demographic characteristics was performed to estimate the focal relations between overall measures.

Second, the LPA was carried out. There were two separate analyses that were performed to establish parent work addiction and demanding organization profiles. The first analysis was performed with two scales (work addiction of mother and father) and the second one was carried out with six scales (family-unsupportive organizational environment, competitive organizational climate, work addiction of a manager, workload, interpersonal conflicts at work and work role conflict) as input variables. A series of LPA models with each analysis were tested to determine which model provided the best fit. We started with the solution of one profile and successively added an additional profile until the final model was made. We stopped generating models after two models in a row had no longer gained statistical significance. We generated a total of eight models for parent work addiction and five models for demanding organization. Based on the recommendations in the literature [75], the optimal number of profiles (i.e., the best-fitting model) was determined using several criteria: Consistent Akaike’s Information Criterion (CAIC), Bayesian Information Criterion (BIC), Approximate Weight of Evidence Criterion (AWE), and Lo–Mendell–Rubin Adjusted Likelihood Ratio Test (LMR-A-LRT). The best possible model had to have lower CAIC, BIC, AWE values and statistically significant LMR-A-LRT test (*p* < 0.05) [75]. We also examined the entropy, which refers to the accuracy of assigning people to profiles; higher entropy values indicate greater accuracy (i.e., >0.80 denotes high classification quality, from 0.50 to 0.80 shows mediocre classification quality, and ≤0.50 indicates poor classification quality [76]). In addition to fit criteria, we evaluated interpretability of the latent profiles and also assessed if there were enough people in each profile (preferably more than 5% of the sample based on practices of other studies [77]). The parameters of the models were estimated using maximum-likelihood estimation with robust standard errors (MLR). 

The final stage consisted of hypotheses testing. The latent variable SEM was conducted to test H1 and H2. A single mediation model was created using a full sample. To test H3 (a, b) and H4 (a, b), a multi-group path analysis with SEM was carried out. It was performed in two stages. In the first stage, separate models for parent work addiction profiles and demanding organization profiles as potential moderators were generated where relationships (see H1 and H2) were assumed to be equal for employees across the established profiles. In the second stage, we generated another two models in which relationships were assumed not to be equal for employees across the established profiles. The models in the second stage were constructed to test both conditional direct and indirect effects. To define confidence intervals when testing all hypotheses, the bias-corrected bootstrap method was used. In total, 10,000 bootstrapping samples were generated by random sampling. Proof of a significant estimate was obtained if the confidence interval of 95% did not contain zero. In testing the hypotheses, the participants’ socio-demographic characteristics were controlled. The parameters were estimated using maximum-likelihood (ML) estimation. 

## 3. Results

### 3.1. Confirmatory Factor Analysis, Validity and Reliability

The CFA model comprising all the variables of the current study presented an acceptable fit to the data: χ^2^ (4268) = 12,408.92, *p* < 0.001, CFI = 0.94; TLI = 0.92; RMSEA = 0.05. Table 1 shows the estimates indicating reliability and validity of the overall measures. All items in the model displayed acceptable standardized loadings [78], which ranged from 0.40 to 0.90. Cronbach’s alpha values ranged from 0.78 to 0.93; all exceeding the recommended threshold of 0.70 [79]. Composite reliability (CR) values also exceeded the recommended value of 0.70 [78] (i.e., ranged from 0.79 to 0.93). In the present study, there were some cases that failed to meet the recommended threshold of ≥ 0.50 for Average Variance Extracted (AVE) values [78]. However, the threshold for AVE slightly lower than 0.50 can be accepted if CR is high enough for the particular construct [80]. Hence, considering all the values presented in Table 1, we conclude that reliability and convergent validity of the instruments are confirmed. 

We used heterotrait-monotrait (HTMT) ratio values [81] in order to test discriminant validity of the constructs. The results (see Table 1) showed that the values were below the recommended threshold (i.e., 0.90 [81]). Therefore, discriminant validity has also been established.

### 3.2. Test of Common Method Variance

Since our data were self-reported, we aimed to assess the extent to which common method variance was a problem in the current study. For that purpose, the Harman’s single-factor test [82] was conducted. We constructed a one-factor model where all items were loaded on a single latent variable and found that data fit to such model was poor: χ^2^ (4329) = 27,838.29; *p* < 0.001; CFI = 0.39; TLI = 0.37; RMSEA = 0.09.

### 3.3. Correlations and Descriptive Statistics for Study Variables

To establish a link between the study variables, a partial correlation was calculated. The results revealed most of the study variables to be significantly positively intercorrelated. The summary of the correlation coefficients, as well as the means and standard deviations (SD), is shown in Table 2.

### 3.4. Latent Profile Analysis

#### 3.4.1. Comparison of Latent Profile Models

LPA analysis for parent work addiction was first performed. Following the recommendations [75], we primarily relied on the CAIC, BIC and AWE when determining how many profiles to retain. However, the evidence for the best-profile model was rather ambiguous: the CAIC reached a minimum at the five-profile model, the BIC reached a minimum at the six-profile model, the AWE reached a minimum at the two-profile model. We further performed the LPA analysis for demanding organization. The evidence for the best profile solution was ambiguous in this case too. The CAIC and BIC values kept decreasing with models characterized by a higher count of latent profiles, whereas the AWE reached a minimum at the three-profile model (for detailed fit values see Table 3). 

As neither the CAIC, BIC, nor AWE provided clear guidance in the model selection process in both LPA cases, we used a previously recommended [83] graphical representation (i.e., the elbow plot) of these fit criteria when determining the final solution. In the case of parent work addiction, there was a clear elbow for the AWE index at the two-profile model. The elbow for the CAIC and BIC indices were also mostly supportive for the two-profile solution. In the case of the demanding organization, there was a visible elbow at the three-profile model for all analyzed indices. Hence, based on the elbow plot results, statistically significant LMR-A-LRT tests (see Table 3), acceptable classification quality (see Table 3), a sufficient number of people in each profile (see notes of Table 4), and interpretability of the latent profiles, we retained a two-profile solution as our final model for parent work addiction and a three-profile solution as our final model for a demanding organization. 

#### 3.4.2. Description of Established Latent Profiles

Table 4 shows mean levels of generated profiles (i.e., two profiles for parent work addiction and three profiles for a demanding organization). The first profile of parent work addiction showed lower work addiction scores for father and mother. We labeled this profile as less addicted parents. The second profile of parent work addiction showed higher work addiction scores for both mother and father. We labeled this profile as more addicted parents. 

The first profile of a demanding organization was characterized by the lowest scores for all the variables analyzed. We labeled this profile as a slightly demanding organization. The second profile was characterized by moderate scores for five variables (i.e., family-unsupportive organizational environment, work addiction of a manager, workload, interpersonal conflicts at work, work role conflict) and the highest scores for one variable (i.e., competitive organizational climate). We labeled this profile as a moderately demanding organization. The third profile was characterized by the highest scores for five variables (i.e., family-unsupportive organizational environment, work addiction of a manager, workload, interpersonal conflicts at work, work role conflict) and the moderate scores for one variable (i.e., competitive organizational climate). We labeled this profile as a highly demanding organization.

### 3.5. Hypothesis Testing

#### 3.5.1. Mediation Analysis

The fit indices for the mediation model with the full sample were acceptable: χ^2^ (654) = 1443.89, *p* < 0.001, CFI = 0.95; TLI = 0.93; RMSEA = 0.04. In a mediation model, perfectionism was unrelated to work addiction (*β* = 0.04, *p* > 0.05); type A personality was related to work addiction significantly positively (*β* = 0.69, *p* < 0.001). The indirect effect of perfectionism on work addiction via extrinsic motivation was positive and significant (indirect effect = 0.04, 95% CI = [0.004, 0.080]). The indirect effect of type A personality on work addiction was insignificant (indirect effect = −0.01, 95% CI = [−0.042, 0.009]).

#### 3.5.2. Moderated Mediation Analysis

Finally, the moderation aspect was added. Following the hypotheses (H3 (a, b) and H4 (a, b)), only high- and low-level situational factors’ profiles were assessed when carrying out the moderation analysis. As it was expected, the initial multi-group SEM models (with constraints) were not supported. Fit indices for the model that tested moderating effects of parent work addiction profiles were χ^2^ (8) = 60.90, *p* < 0.001, CFI = 0.88; TLI = 0.85; RMSEA = 0.15 and for the model that tested moderating effects of demanding organization profiles were χ^2^ (13) = 57.65, *p* < 0.001, CFI = 0.89; TLI = 0.87; RMSEA = 0.13. This indicated that moderating effects might actually exist and, therefore, the subsequent two models (with no constraints) were generated. The model that tested moderating effects of parent work addiction profiles showed df = 0 and was identified as saturated; the model that tested moderating effects of demanding organization profiles showed good fit to data (χ^2^ (3) = 6.41, *p* = 0.09, CFI = 0.99; TLI = 0.96; RMSEA = 0.05). We analyzed these models in more detail (for results see Table 5).

It was found (see Model 1 in Table 5) that parent work addiction profiles did not moderate the direct relationships between employee perfectionism, type A personality, and work addiction. However, the moderating effect held for the indirect links. Indirect relationships between perfectionism, type A personality, and work addiction via extrinsic motivation were positive and stronger for employees in a high-level (i.e., more addicted parents) profile. It was further found (see Model 2 in Table 5) that direct relationships between employee perfectionism, type A personality, and work addiction were moderated by demanding organization profiles. These relationships were positive and stronger for employees in a high-level (i.e., highly demanding organization) profile. However, demanding organization profiles did not moderate indirect relationships between employee perfectionism, type A personality, and work addiction. 

## 4. Discussion

Work addiction is a complex phenomenon and, thus, different sources of influences collectively determine whether one becomes addicted to work [24]. Therefore, a reliable picture of the origin of work addiction could only be presented by analyzing multiple factors. Against this background, the present research extended the traditional approach that focused on dispositions as precursors of work addiction by focusing on a rather neglected area—how individual interacts with the situation in predicting work addiction. In a preparatory stage, LPA was performed. The results were largely expected since they revealed quantitatively different situational factors’ profiles and aligned in this way with the general tendencies of studies in similar fields (e.g., [67,68]). This allowed us to move on to hypothesis testing (for summary see Table 6). 

Both H1 and H2 were partially verified. The results obtained were not fully in line with the results and observations found in existing literature (e.g., [8,56]) since perfectionism and work addiction were only related through indirect links (mediated by motivational forces), whereas type A personality acted only as a direct predictor of work addiction. At first sight, it seems that extrinsic motivation could be a factor that fully explains the perfectionism–work addiction link and that it may be worth testing some other agents rather than motivational ones in order to clarify if there are other elements that could explain the relationship between type A personality and work addiction. Further, moderators were added to our analysis. Direct relationships between perfectionism, type A personality, and work addiction were found to be positive and stronger for the employees working in highly demanding organizations. However, parent work addiction profiles did not moderate these direct links. This enabled us to confirm H3b but not H3a. In support of H4a, indirect relationships between perfectionism, type A personality, and work addiction (via extrinsic motivation) were positive and stronger for the employees having parents with a higher level of work addiction. However, demanding organization profiles did not moderate these indirect links, thus precluding us from confirming H4b. With a few exceptions, these findings are in line with the presumption of Liang and Chu [17], that working in demanding organizations and having work-addicted parents produce a combined effect with certain personal factors in such a way that the risk of work addiction increases. However, in a very general sense, it seems that the fact of having addicted parents is more relevant to extrinsic motivation as compared to the fact of working in demanding organizations because indirect effects (via extrinsic motivation) were moderated only by parent work addiction profiles. This may be due to the conditional acceptance of such parents, which actually shapes a child’s motivational background [64]. Hence, it is likely that extrinsic motivation is simply more easily activated when there are work-addicted parents. It could also be assumed that the fact of working in a demanding organization is more relevant to the expression of certain personal characteristics (such as achievement orientation, competitiveness, exactingness, etc.). This may explain why direct relationships were moderated only by demanding organization profiles. 

To sum up, the results indicate that the context in which the links among the perfectionism, type A personality, extrinsic motivation, and work addiction are evaluated may determine the strength of these links. This obviously calls for a broad viewpoint and careful consideration of situational moderators, which we see as an advantage of the present study. However, this study in no way suggests that personality, family, or organization alone are unable to evoke work addiction; each of these sources of influences by itself is actually of importance. Rather, our message is that the greatest risk of becoming addicted comes from the entirety of factors. It is multiple factors as a whole that create the most conductive circumstances for work addiction to arise. Therefore, instead of focusing on several factors of the narrow effects, we suggest that researchers should take a more extensive viewpoint in future studies with integrated new constructs and ideas.

### 4.1. Theoretical Contribution

This paper contributes to the theory in several respects. First, our study presented a comprehensive picture of how work addiction may develop by testing an integrated model of work addiction comprised of all sources of influences that have been recognized to be major ones in predicting this phenomenon (i.e., personality, organization, family) [20]. This is not necessarily surprising, but it does provide empirical support for what have been largely only theoretical models in the field of work addiction.

Furthermore, we used a previously unexplored model of Liang and Chu [17] as the basis for formulating our hypotheses. We tested this model in all its parts and provided empirical evidence for originally proposed linkages between precursors of work addiction. The present study also updated the model by adding new variables and links. By doing so, we offered some new insights and took a step towards a more nuanced framework of work addiction precursors.

The LPA analysis and a person-centered perspective is also a contribution. The use of the person-centered approach fills the gap in literature, which is dominated by the variable-centered approach that overlooks the configurations of precursors of work addiction. At the same time, it extends our understanding of possible methods for investigating factors determining work addiction. 

### 4.2. Practical Implications

Our study is also valuable as a basis for intervention since the results obtained tell us what to focus on when solving the issue of work addiction. Increasing awareness of stimulating factors (of personal cues for addictive behaviors in the first place) may be a primary tool opening up ways to intervening in the addiction process. It lays the groundwork for voluntary control and thereby helps to avoid falling back into the trap of addiction [84]. This task is hard to implement, though, as it may require a persistent intervention, a lot of effort, and reflective skills. However, many treatment techniques have been developed. Cognitive behavioral therapy was proposed to be the main treatment method for work addiction [85].

The idea that work addiction is solely a personal problem was not supported by evidence, though. Therefore, it might be worthwhile to focus on external conditions too, especially given the fact that they are easier to change as compared to relatively inflexible personal factors [20]. As regards organizations, all practices that reward excessive work behavior should be modified. Managers play a leading role here, mainly because their behavior and attitudes are directly transmitted into the organizational practices. Thus, an effective intervention in discouraging employee work addiction by modifying organizational practices would only be successful when management acts as a role model of normal working behavior (e.g., by displaying behaviors that favor a healthy work–life balance and minimize overwork) [86]. This may initiate a reinterpretation of the overall organizational environment and stimulate employees changing their unhealthy working habits. 

The findings of the present study indicate that parents are also of significance. Therefore, we would like to convey a message to the families that face work addiction issues. To apply for family therapy could be very useful, so that parents become aware of how they may model the behavior with their own example and reinforce work addiction in a child or an adult child. Counselors, in turn, should instruct work-addicted parents to monitor their own working behavior and to show unconditional regard for their children as opposed to measuring the child’s worth by his/her performance or by what he/she achieves [87].

### 4.3. Limitations 

This study has several limitations, and its results should be interpreted with caution. The first limitation refers to the obscurity surrounding the focal phenomenon of the present study. There is little doubt that work addiction does exist [88,89]. Nevertheless, it has not been formally recognized yet [90]. More evidence and clarity (especially regarding the overlapping phenomena) are still needed. Therefore, the prime goal of future studies should be oriented to solving conceptualization issues related to work addiction. 

Further, the study utilized a cross-sectional research design for data collection, which is held in low esteem despite its widespread use. It is generally considered that findings of a cross-sectional study indicate no causality. It should be noted, therefore, that the causal language used throughout this paper is a statistical expression as our results do not have a valid basis for making causal inferences. It is also generally accepted that the longitudinal design offers considerable advantages over a cross-sectional design in this respect. However, an often-overlooked fact is that the study using a cross-sectional design can provide evidence for all elements on which the causal case is built and that a longitudinal design is not necessarily superior in determining causation, especially when considering the way such designs are usually applied (for more information see [91]). Hence, cross-sectional designs are not as anemic as many would believe or considerably weaker than longitudinal designs [91]. The present study had full potential to tell us much of value, it was helpful in testing explanatory mechanisms, and it provided a solid basis for moving forward and going beyond what the cross-sectional design can do.

Furthermore, the hypothesized predictors and criterion variable were obtained from the same rater. An important limitation is that neither parents nor managers directly participated in the study; instead, the participants themselves provided answers about their parents’ and managers’ behaviors. Therefore, common method variance could have biased the results. We addressed this limitation, in some respect, with diagnostic techniques (i.e., the Harman’s single-factor test [82]) that are dedicated to indicating whether a single factor accounts for all of the covariances among the items. The fit of a single-factor model was poor, which implies that common method variance is not a major problem in the present study. However, such possibility cannot be dismissed entirely [82]. Therefore, upcoming studies employing alternative methods, such as parents’, supervisors’, or co-workers’ reports, are needed to draw more solid conclusions. Running statistical procedures that directly control the effects of common method variance is also an effective remedy. 

The participants in this study were all Lithuanians, so there may also be some geographical and cultural limitations related to the generalization of the findings. Since some of our constructs may be culturally and/or geographically sensitive (i.e., work addiction), it would be useful for future research to replicate this study within other contexts. In addition, the data in this study were collected using a non-representative sampling method. Furthermore, the sample was not well-balanced in terms of socio-demographic characteristics since the participants were predominantly females with higher education. Moreover, the participants were quite young. Finally, participation in the study was voluntary without any inducements, which might have resulted in a sample bias toward employees who were more work addicted. All this implies that even though the sample size was quite large, our findings could not be broadly applied.

Another limitation is that the highly demanding organization profile was rather small in terms of the number of participants. Although we followed previous practices for the profiles sizes when conducting the LPA [77]; however, these sizes were not necessarily sufficient for a subsequent multi-group analysis. It may have biased the estimates or standard errors and should be considered when interpreting our results. 

Moreover, despite the fact that the study considered all main sources of influences explaining work addiction (i.e., personality, organization, family) [20], a set of variables included remained limited in certain respects. For instance, the variable of type A personality did not incorporate the personality construct by itself, since other personality types were not regarded in the study as a counterpoint, etc. Hence, it would be misleading to conclude that the sources of influences analyzed were fully explored. Rather, only some of the factors (albeit the main ones in the field of work addiction) comprising these sources were investigated. Furthermore, the consequences were not included in a research model. For a more integrated and complete picture of work addiction, we suggest that possible outcomes should be taken into consideration.

Finally, it is noteworthy that our study was conducted under specific circumstances (i.e., during the COVID-19 pandemic). The pandemic affected most aspects of everyday life, including work patterns, social interactions, etc. [92], and may have had an effect on our findings too. Therefore, the recommendation for future research is to conduct the study once again during the post-pandemic situation and to delve into arising differences. This would complement the research line with the comparison of the examined relations in two different situations.

## 5. Conclusions

In a broad sense, this study aimed to improve the understanding of the linkages between an individual and a situation in the field of work addiction. Supporting our main assumptions, we found out that the relationships between personal factors analyzed and work addiction actually show situational specificity in terms of strength of these relationships. The present study makes a theoretical and practical contribution and opens up an opportunity to continue the research line with other methods of data collection and analysis, utilizing more powerful samples and including more constructs. 

## Figures and Tables

**Table 1:** Reliability and validity of instruments.

Variables	HTMT Ratio											Standardized Factor Loadings	AVE	CR	Cronbach’s Alpha
	1	2	3	4	5	6	7	8	9	10	11				
1. Work addiction of employee												0.41–0.85	0.51	0.87	0.85
2. Extrinsic work motivation	0.40											0.40–0.83	0.49	0.90	0.91
3. Perfectionism	0.52	0.57										0.56–0.90	0.54	0.92	0.92
4. Type A personality	0.65	0.46	0.70									0.41–0.87	0.45	0.89	0.81
5. Work addiction of mother	0.30	0.15	0.25	0.27								0.74–0.86	0.64	0.93	0.93
6. Work addiction of father	0.25	0.14	0.35	0.32	0.43							0.72–0.86	0.65	0.93	0.93
7. Family-unsupportive organizational environment	0.26	0.04	0.24	0.31	0.18	0.26						0.43–0.79	0.49	0.91	0.85
8. Competitive organizational climate	0.37	0.08	0.34	0.38	0.21	0.23	0.38					0.54–0.80	0.49	0.79	0.78
9. Work addiction of manager	0.28	0.20	0.24	0.25	0.28	0.26	0.24	0.29				0.59–0.84	0.54	0.89	0.89
10. Workload	0.46	0.12	0.40	0.61	0.20	0.27	0.30	0.34	0.21			0.78–0.87	0.67	0.91	0.91
11. Interpersonal conflicts at work	0.08	0.20	0.11	0.28	0.08	0.14	0.38	0.38	0.09	0.42		0.51–0.89	0.56	0.83	0.81
12. Work role conflict	0.41	0.09	0.33	0.42	0.27	0.27	0.45	0.49	0.19	0.47	0.45	0.60–0.82	0.50	0.89	0.89

**Table 2:** Correlations, means, and standard deviations of the study variables.

Variables	Mean	SD	1	2	3	4	5	6	7	8	9	10	11
1. Work addiction of employee	2.81	0.85											
2. Extrinsic work motivation	4.27	1.26	0.27 ***										
3. Perfectionism	4.36	1.29	0.43 ***	0.51 ***									
4. Type A personality	2.55	0.61	0.53 ***	0.36 ***	0.57 ***								
5. Work addiction of mother	2.82	0.99	0.26 ***	0.13 **	0.22 ***	0.23 ***							
6. Work addiction of father	2.73	1.02	0.23 ***	0.13 **	0.30 ***	0.25 ***	0.41 ***						
7. Family-unsupportive organizational environment	2.61	0.64	0.22 ***	−0.06	0.20 ***	0.27 ***	0.17 ***	0.25 ***					
8. Competitive organizational climate	3.44	1.40	0.31 ***	0.06	0.28 ***	0.33 ***	0.17 ***	0.19 ***	0.31 ***				
9. Work addiction of manager	2.77	0.92	0.23 ***	0.17 ***	0.20 ***	0.20 ***	0.27 ***	0.25 ***	0.21 ***	0.25 ***			
10. Workload	2.95	1.04	0.38 ***	0.08 *	0.32 ***	0.50 ***	0.17 ***	0.26 ***	0.27 ***	0.29 ***	0.18 ***		
11. Interpersonal conflicts at work	1.61	0.71	0.11 **	−0.11 **	0.14 ***	0.27 ***	0.07	0.13 **	0.34 ***	0.32 ***	0.11 **	0.39 ***	
12. Work role conflict	3.46	1.40	0.35 ***	0.08	0.30***	0.38 ***	0.24 ***	0.24 ***	0.39 ***	0.40***	0.18 ***	0.44 ***	0.39 ***

* *p* ≤ 0.05, ** *p* ≤ 0.01, *** *p* ≤ 0.001.

**Table 3:** Fit indices, entropy, and comparison of estimated latent profile models.

Model	LL	npar	CAIC	BIC	AWE	Entropy	LMR-A-LRT (*p*)
Parent work addiction latent profile models							
1 class	−4186.887	4	8403.499	8399.498	8437.225	1.000	
2 class	−4133.184	7	8318.387	8311.388	8377.407	0.698	0.0000
3 class	−4114.821	10	8303.955	8293.956	8388.269	0.680	0.0201
4 class	−4098.159	13	8292.925	8279.926	8402.533	0.641	0.1762
5 class	−4082.654	16	8284.209	8268.210	8419.111	0.714	0.0016
6 class	−4071.807	19	8284.809	8265.809	8445.005	0.729	0.0108
7 class	−4064.382	22	8292.253	8270.253	8477.743	0.767	0.3438
8 class	−4054.033	25	8293.849	8268.850	8504.633	0.826	0.1704
Demanding organization latent profile models							
1 class	−11,884.617	12	23,858.410	23,846.411	23,959.586	1.000	
2 class	−11,613.587	19	23,368.369	23,349.369	23,528.565	0.761	0.0000
3 class	−11,494.567	26	23,182.349	23,156.349	23,401.563	0.827	0.0001
4 class	−11,461.194	33	23,167.622	23,134.622	23,445.856	0.844	0.2321
5 class	−11,424.798	40	23,146.849	23,106.850	23,484.102	0.852	0.0582

Note: LL = Log likelihood; npar = number of free parameters; CAIC = Consistent Akaike’s Information Criterion; BIC = Bayesian Information Criterion; AWE = Approximate Weight of Evidence Criterion; LMR-A-LRT (*p*) = *p* value for Lo–Mendell–Rubin Adjusted Likelihood Ratio Test.

**Table 4:** Description of the latent profiles.

Variables	Established Profiles		
	Parent work addiction profiles		
	Profile 1: Less addicted parents		Profile 2: More addicted parents
Work addiction of mother	13.467		23.558
Work addiction of father	14.872		21.661
	Demanding organization profiles		
	Profile 1: Slightly demanding organization	Profile 2: Moderately demanding organization	Profile 3: Highly demanding organization
Family-unsupportive organizational environment	33.258	41.130	44.767
Competitive organizational climate	11.505	17.224	16.977
Work addiction of manager	17.618	19.113	22.376
Workload	12.389	18.361	18.857
Interpersonal conflicts at work	5.152	7.385	14.823
Work role conflict	22.244	35.676	38.212

Note: Two-profile solution for parent work addiction (Profile 1 (n = 222, 35.7%), Profile 2 (n = 399, 64.3%)); three-profile solution for demanding organization (Profile 1 (n = 389, 62.6%), Profile 2 (n = 198, 31.9%), Profile 3 (n = 34, 5.5%)).

**Table 5:** Results of multi-group SEM models.

	Model 1 (Testing Moderating Effects of Parent Work Addiction Profiles)	Model 2 (Testing Moderating Effects of Demanding Organization Profiles)
Relationships	Estimates [95% CI]	Estimates [95% CI]
Perfectionism with work addiction (LP)	0.112 [0.007; 0.215]	0.058 [−0.007; 0.128]
Perfectionism with work addiction (HP)	0.106 [0.039; 0.175]	0.402 [0.143; 0.791]
Type A personality with work addiction (LP)	0.679 [0.485; 0.870]	−0.079 [−0.856; 0.445]
Type A personality with work addiction (HP)	0.446 [0.299; 0.592]	0.592 [0.450; 0.731]
Perfectionism with work addiction via extrinsic motivation (LP)	−0.021 [−0.076; 0.033]	0.044 [0.009; 0.078]
Perfectionism with work addiction via extrinsic motivation (HP)	0.049 [0.023; 0.083]	0.066 [−0.251; 0.322]
Type A personality with work addiction via extrinsic motivation (LP)	−0.005 [−0.047; 0.007]	0.027 [0.005; 0.068]
Type A personality with work addiction via extrinsic motivation (HP)	0.045 [0.015; 0.092]	−0.041 [−0.471; 0.120]
Moderating effect of established profiles on direct link between perfectionism and work addiction	0.007 [−0.118; 0.125]	−0.344 [−0.731; −0.075]
Moderating effect of established profiles on direct link between type A personality and work addiction	0.233 [−0.006; 0.479]	−0.671 [−0.126; −1.443]
Moderating effect of established profiles on indirect link between perfectionism and work addiction via extrinsic motivation	−0.070 [−0.136; −0.011]	−0.022 [−0.285; 0.296]
Moderating effect of established profiles on indirect link between type A personality and work addiction via extrinsic motivation	−0.050 [−0.106; −0.016]	0.067 [−0.105; 0.493]

Note: LP = low-level profile; HP = high-level profile; moderating effects were calculated by subtracting HP from LP.

**Table 6:** Summary of hypothesis testing.

Hypotheses	Outcome
**H1:** *Employee perfectionism and type A personality are related to increased work addiction.*	Partially confirmed
**H2:** *Employee perfectionism and type A personality are related to increased work addiction through higher levels of extrinsic work motivation.*	Partially confirmed
**H3a:** *Direct relationships between employee perfectionism, type A personality, and work addiction are moderated by parent work addiction profiles. These relationships are stronger for employees in a high-level profile (as compared to those in a low-level profile).*	Rejected
**H3b:** *Direct relationships between employee perfectionism, type A personality, and work addiction are moderated by demanding organization profiles. These relationships are stronger for employees in a high-level profile (as compared to those in a low-level profile).*	Confirmed
**H4a:** *Indirect relationships between employee perfectionism, type A personality, and work addiction via extrinsic work motivation are moderated by parent work addiction profiles. These indirect relationships are stronger for employees in a high-level profile (as compared to those in a low-level profile).*	Confirmed
**H4b:** *Indirect relationships between employee perfectionism, type A personality, and work addiction via extrinsic work motivation are moderated by demanding organization profiles. These indirect relationships are stronger for employees in a high-level profile (as compared to those in a low-level profile).*	Rejected

## Data Availability

The data analyzed in this study may be obtained from the corresponding author upon reasonable request.
